# Cold cabbage application and postnatal mothers' perceptions of breast engorgement: A mixed method study

**DOI:** 10.6026/9732063002002034

**Published:** 2024-12-31

**Authors:** Christena P, Elizebeth Rani V, Pushpa S, Banusri J

**Affiliations:** 1 Department of Obstetrics and Gynecology Nursing, Sri Balaji Vidyapeeth, Deemed to be University, Puducherry & KMC College of Nursing, Trichy, India; 2 Department of Obstetrics and Gynecology Nursing, VHS-M.A.Chidambaram College of Nursing, Chennai, India; 3 Department of Obstetrics and Gynecology Nursing, Dhanalakshmi Srinivasan College of Nursing, Perambalur, India; 4 Department of Nursing, KMC College of Nursing, Trichy, India

**Keywords:** Breast engorgement, cold cabbage leaves, postnatal mothers, breastfeeding, natural remedy, quasi-experimental study

## Abstract

A study explored the effectiveness of cold cabbage leaf application in reducing pain and engorgement among postnatal mothers using a mixed-methods approach with 40 participants. The treatment involved applying cold cabbage leaves for 30 minutes, three times a day for three days, which resulted in significant reductions in both pain (from 4.9 to 2.9) and engorgement (from 4.45 to 2.8) at p < 0.001. Qualitative interviews with 10 mothers revealed that many felt relief from pain and swelling and appreciated the simplicity, affordability and natural aspects of the remedy. However, some mothers experienced discomfort from the coldness and difficulty keeping the cabbage leaves in place. Despite these challenges, the majority were satisfied with the treatment and preferred it over medications. The study suggests that cabbage leaf application is an effective, low-cost solution for managing breast engorgement, with potential benefits for breastfeeding, although future research could focus on improving practical application and providing clearer instructions.

## Background:

Breast engorgement is a common and painful condition experienced by new mothers, characterized by swollen, painful breasts due to excess milk production [1]. It often occurs in the first two weeks postpartum and can make breastfeeding difficult [2]. Untreated, engorgement can lead to complications like blocked ducts and mastitis, potentially causing early cessation of breastfeeding [3, 4]. Various remedies are used to alleviate the discomfort, including the popular but debated use of cold cabbage leaves. Some studies show cabbage leaves may reduce inflammation and improve milk flow, while others report minimal effects [5, 6]. Therefore this study aims to evaluate the effectiveness of cabbage leaf treatment on breast engorgement and understand mothers' perceptions of this remedy and also to explores how cabbage leaf application, along with lactation management education, can improve breastfeeding outcomes and reduce the impact of engorgement.

## Methodology:

A quasi-experimental, mixed-method research approach with an explanatory sequential design was employed for this study, which used a pre-experimental one-group pre-test-post-test design to evaluate the effectiveness of cold cabbage leaf application on breast engorgement among postnatal mothers. The study was conducted at Krishnaveni Maternity Hospital, Thuraiyur, with a sample size of 40 postnatal mothers. Participants were selected based on inclusion criteria: they experienced breast engorgement, were within the first 5 days of the puerperium period, were primi or multi-gravida, spoke and understood Tamil or English and were willing to participate. Exclusion criteria included mothers receiving lactation suppressants, those with breast complications (such as mastitis, abscess, or cracked nipples), allergies to sulfa or cabbage and those unwilling to participate. The sample size of 40 was calculated using a standard formula for pre-test-post-test designs, ensuring a sufficient power to detect differences in the effectiveness of the cabbage leaf intervention, accounting for an expected moderate effect size and a 95% confidence level with a 5% margin of error.

The data collection tool was divided into three sections:

[1] Section A: Background information, which included demographic and obstetric details of the participants.

[2] Section B: A Numerical Rating Scale (NRS) to assess breast pain in postnatal mothers. This standardized tool uses a 10-point scale to classify breast pain into four categories: no pain, mild pain, moderate pain and severe pain.

[3] Section C: A modified four-point scale to assess the severity of breast engorgement in postnatal mothers. The scale classifies engorgement into four levels: no engorgement, mild engorgement, moderate engorgement and severe engorgement.

## Ethical consideration:

The study received ethical approval from the Institutional Ethics Committee (IEC), to ensure compliance with ethical standards in research involving human subjects. Informed consent was obtained from all participants and confidentiality was maintained throughout the study.

## Data collection procedure:

## Phase - 1 quantitative phase:

On the first day, the investigator introduced her to the postnatal mothers and explained the purpose of the study. After obtaining informed consent, the investigator assessed the effectiveness of cold cabbage leaf application on breast engorgement. Two postnatal mothers were selected each day for the study. A pretest was conducted and then cold cabbage leaves were placed inside the mothers' brassieres for 30 minutes, three times a day before breastfeeding, for three consecutive days. After the intervention, the level of breast pain and engorgement was reassessed using the Numerical Rating Scale and the Modified Four-Point Breast Engorgement Scale. The collected data were analyzed using descriptive and inferential statistics.

## Results and Discussion:

## Demographic variables:

Most postnatal mothers were aged 24-29 years (40%), with the majority being Hindu (48%), having completed secondary education (35%) and being unemployed (80%). In terms of income, 38% earned between Rs 5000-10,000 and 45% lived in rural areas. Most mothers (70%) came from nuclear families.

## Obstetrical variables:

Most postnatal mothers were primi gravida (55%) and had a Caesarean section (60%). The majority initiated breastfeeding 1-2 hours after delivery, with most breastfeeding for less than 5 minutes per breast. About 45% of mother's breastfed every 2 hours and 53% practiced mixed feeding. Most had no history of breast engorgement (90%).

## Comparison of pre-test and post-test level of pain and breast engorgement among postnatal mothers:

**Figure 1:**
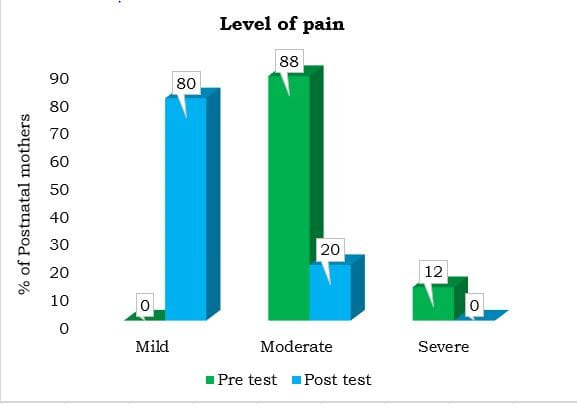
Comparison of pretest and post-test level of pain

**Figure 2:**
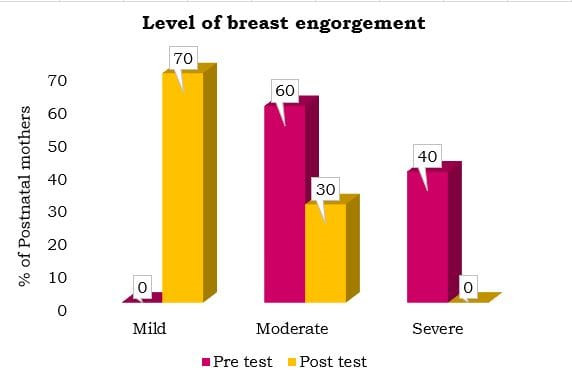
Comparison of pretest and post-test Level of Breast Engorgement

This study shows that cabbage leaf application effectively reduces pain and breast engorgement, while also improving breastfeeding outcomes in postnatal mothers. Figure 1 & Figure 2 shows that, in the pretest, all mothers had severe pain and 60% had severe engorgement. After using cabbage leaves for one day, 80% reported mild pain and 70% had mild engorgement. Additionally, 60% of mothers showed improved breastfeeding. The statistical analysis (t = 23.472, p < 0.001) confirmed that cabbage leaf therapy significantly reduced pain and engorgement. These results are similar to those of Dhoom, Sushmaben, Maradiya, Janaki and Doss (2024), which also found cabbage leaves to be effective in improving breastfeeding and alleviating breast engorgement [7, 9 & 10].

The results of this study, Table 1 demonstrate that applying cold cabbage leaves significantly reduced pain and breast engorgement in postnatal mothers. Pre-treatment, the average pain level was 4.9 (SD = 1.194), which dropped to 2.9 (SD = 1.1277) post-treatment, while breast engorgement decreased from an average of 4.45 (SD = 1.460) to 2.8 (SD = 0.7974), with both changes showing statistically significant differences (p < 0.001). These findings align with the study by Sushmaben *et al.* (2024), who also found that cabbage leaf application significantly reduced pain and engorgement among postnatal mothers [8]. In their study, 100% of participants had severe pain and 60% had severe engorgement at baseline, but after the intervention, both pain and engorgement levels dropped significantly and breastfeeding outcomes improved. The statistical significance (t = 23.472, p < 0.001) in both studies confirms the effectiveness of cabbage leaf therapy. This non-pharmacological, low-cost intervention appears to be an effective solution for managing breast engorgement and supporting breastfeeding, ultimately improving maternal comfort and enhancing the breastfeeding experience [13, 15].

## Association between post-test level of pain and breast engorgement with the demographic and obstetrical variables of postnatal mothers:

There was a significant association between post-test pain levels and gravida, parity and breastfeeding practices (initiation, duration and frequency). Similarly, the duration of breastfeeding in each breast was significantly associated with post-test breast engorgement levels [9, 11, 12 & 14].

## Phase 2: qualitative phase:

In Phase 2 of the study, qualitative data were collected through in-depth, semi-structured interviews with a subset of 10 postnatal mothers who had participated in the quantitative phase. Participants were purposively selected to represent a range of experiences with cabbage leaf application (*e.g.*, very satisfied, somewhat satisfied, dissatisfied). The inclusion criteria for selection were mothers who had experienced breast engorgement and used the cabbage leaf treatment as part of the study. Purposive sampling was employed to ensure diversity in experiences and the sample size of 10 was based on the principle of data saturation, where no new information was expected to emerge from further interviews. Semi-structured interviews were guided by open-ended questions exploring participants' experiences with breast engorgement, their perceptions of the cabbage leaf treatment and their overall satisfaction with the remedy. The interviews were audio-recorded with consent and transcribed verbatim for thematic analysis.

## Findings of the qualitative data:

The qualitative analysis of the in-depth interviews revealed six key themes and sub-themes, reflecting the postnatal mothers' experiences with cabbage leaf treatment for breast engorgement. Below are the detailed themes and their respective sub-themes, each supported by participant excerpts.

## Theme 1: Relief from pain and swelling:

Most participants reported significant relief from pain and breast swelling after using cabbage leaves. This theme underscores the perceived effectiveness of the remedy in alleviating the discomfort associated with breast engorgement.

## Sub-theme 1.1: Immediate relief from pain:

Most mothers reported feeling immediate relief from the pain of engorgement after applying cabbage leaves. The cooling effect was often mentioned as providing soothing comfort and reducing both pain and swelling. One mother said, "When I applied the cabbage leaves, the pain reduced almost instantly. The coolness was such a relief and my breasts felt less full. It helped me relax and focus on breastfeeding" (Mother 1). Another shared, "The pain was terrible before, but after using the cabbage leaves, I could tell it was getting better. My breasts were much softer" (Mother 3). A third mother added, "I felt a huge difference the first time I used it. The cabbage leaves reduced the swelling and pain and I could breastfeed more comfortably" (Mother 5).

## Sub-theme 1.2: Gradual improvement over time:

While some mothers experienced immediate relief, others found the benefits of cabbage leaves took time to show. With continued use over several days, pain and swelling gradually improved. One mother explained, "It didn't work instantly, but after using it for three days, the pain became much more manageable and the swelling went down." (Mother 2), nodding as she recalled her experience. Another mother shared, "At first, I didn't feel much change, but by the third day, my breasts weren't as hard or painful anymore." (Mother 6), smiling as she spoke. A third mother said, "After using it consistently for a few days, the pain faded and I could finally breastfeed without discomfort" (Mother 7), her face relaxing with relief. These responses show that while the relief was gradual, consistent use of cabbage leaves led to noticeable improvement over time.

## Theme 2: Positive perception of the remedy:

Most participants had a positive overall view of the cabbage leaf treatment, appreciating it for its simplicity, affordability and natural composition.

## Sub-theme 2.1: A natural and accessible remedy:

Many mothers appreciated the simplicity and affordability of using cabbage leaves as a natural remedy. One mother said, "I liked that the cabbage leaves were natural and I did not need to rely on medicine. It felt better because it was a natural solution" (Mother 4). Another mother shared, "The best part was that I could do it myself at home without needing to see a doctor or buy expensive products" (Mother 2). A third mother added, "It was so easy. I just put cabbage in my bra and it worked. I liked that it was simple and inexpensive" (Mother 6). These comments show that mothers valued cabbage leaves as an easy, low-cost and natural option for relief.

## Sub-theme 2.2: Satisfaction with the overall treatment:

The treatment was generally perceived as effective, with many participants expressing satisfaction with how it helped reduce pain and swelling. They appreciated that it provided an alternative to pharmacological treatments. Each mother verbalized, "I was really satisfied. The cabbage leaves helped so much and I didn't have to take any pain medicine, which was great" (Mother 8). "It was a relief that I didn't need to go to a clinic or take drugs. The cabbage leaves worked well enough for me" (Mother 9). "I was surprised how effective it was. I thought it might not work, but it really helped with the swelling and made breastfeeding easier" (Mother 10).

## Theme 2: Positive perception of the remedy:

Most participants had a positive overall view of the cabbage leaf treatment, appreciating it for its simplicity, affordability and natural composition.

## Sub-theme 2.1: A natural and accessible remedy:

Several mothers valued the natural and non-medical aspect of cabbage leaves. They were particularly pleased with the idea of using a home remedy, which was both easily accessible and cost-effective. "I liked that the cabbage leaves were natural and I didn't have to rely on medicines. It felt better for me because it was a natural remedy" (Mother 4). "The best part was that it was something I could do myself at home without needing to go to the doctor or buy expensive products" (Mother 2). "It was such an easy thing to do. I just grabbed some cabbage, put it in my bra and it worked. I liked that it was simple and inexpensive" (Mother 6).

## Sub-theme 2.2: Satisfaction with the overall treatment:

The treatment was generally perceived as effective, with many participants expressing satisfaction with how it helped reduce pain and swelling. They appreciated that it provided an alternative to pharmacological treatments. "I was really satisfied. The cabbage leaves helped so much and I didn't have to take any pain medicine, which was great" (Mother 8). "It was a relief that I didn't need to go to a clinic or take drugs. The cabbage leaves worked well enough for me" (Mother 9). "I was surprised how effective it was. I thought it might not work, but it really helped with the swelling and made breastfeeding easier" (Mother 10).

## Theme 4: Personal recommendations and advice:

Mothers who used the cabbage leaf treatment had practical advice for other women considering the remedy.

## Sub-theme 4.1: Importance of consistent uses:

Many participants stressed the importance of consistency, recommending that others apply the cabbage leaves regularly for the best results. "I'd say use them consistently, especially before feeding. It worked better for me when I didn't skip days" (Mother 2)."Need to use them every day, preferably before breastfeeding, for the best results" (Mother 3)."It's really helpful if you stick with it for a few days. That's when you start seeing the results" (Mother 5).

## Sub-theme 4.2: Be cautious of skin sensitivity:

A few mothers warned others about the possibility of skin irritation, advising caution if one has sensitive skin. "I'd say to be careful if you have sensitive skin, because it can cause a rash if you leave the cabbage on too long" (Mother 7). "Make sure to check your skin for irritation, especially if you have delicate skin. I had a small reaction, so I had to be careful" (Mother 8). "If you have sensitive skin, you might want to take the cabbage off after 20 minutes or so to avoid any irritation" (Mother 6).

## Theme 5: Lack of professional support:

This theme highlights the mothers' desire for more professional guidance when using non-pharmacological treatments like cabbage leaves.

## Sub-theme 5.1: Desire for clearer instructions:

Mothers expressed that clearer guidance on how to use cabbage leaves would have been helpful, especially in terms of how long to apply them and when to expect results. "I was not sure how long to leave the cabbage leaves on. It would have been nice to have some guidelines on when to remove them" (Mother 4). "Some instructions from the hospital staff on how to use them properly would have made me feel more confident" (Mother 10). "It would have been helpful to get some tips on how often to use the cabbage leaves. I just figured it out as I went along" (Mother 2).

## Theme 6: Effectiveness of cabbage leaves compared to other remedies:

Mothers were asked to compare cabbage leaf treatment with other remedies they had used or considered for breast engorgement.

## Sub-theme 6.1: Preference for Cabbage Leaves Over Medications:

Many mothers preferred cabbage leaves over medication because of the natural and non-invasive nature of the remedy. They felt it was safer and easier to use, especially during breastfeeding. "I preferred the cabbage leaves because they were natural. I didn't want to take any medication while breastfeeding and it worked well" (Mother 1), "Compared to medications, cabbage leaves felt like a safer choice. It was something I could trust and control myself" (Mother 2). "I didn't want to use any pills because of breastfeeding. The cabbage leaves were a good alternative" (Mother 5).

## Integration of quantitative & qualitative findings:

The integration of quantitative and qualitative findings showed that cold cabbage leaf application significantly reduced pain and engorgement in postnatal mothers. The quantitative data revealed a clear decrease in both pain and engorgement levels, while the qualitative interviews indicated that most mothers felt relief, though some found the coldness uncomfortable and had difficulty keeping the leaves in place. Early and frequent breastfeeding were identified as factors that improved the treatment's effectiveness [4]. Overall, mothers were satisfied with the natural remedy and both the quantitative and qualitative results support the effectiveness of cabbage leaves, while suggesting areas for better practical use.

## Conclusion:

Cold cabbage leaf application effectively reduced pain and breast engorgement in postnatal mothers, providing a natural remedy for postpartum discomfort. The treatment was well-received, though practical challenges highlight areas for improvement in its application.

## Recommendation:

This study highlights the effectiveness of cold cabbage leaf application in relieving breast engorgement and improving postnatal mothers' perceptions. It is recommended that healthcare providers incorporate this cost-effective remedy into postnatal care. Further research should explore its long-term effects and impact on breastfeeding. Nurses and healthcare administrators should consider training programs to educate mothers on the benefits and proper use of cabbage leaf application, enhancing maternal comfort and satisfaction during the postpartum period.

## Funding:

There is no external funding resource

## Figures and Tables

**Table 1:** Effectiveness of cold cabbage leaves application on breast engorgement among postnatal mothers

**Variables**	**Pre test**		**Post test**		**Paired "t" test**
	**Mean**	**SD**	**Mean**	**SD**	
Level of pain	4.9	1.194	2.9	1.1277	7.702 Df - 78 P < 0.001***S
Level of breast engorgement	4.45	1.46	2.8	0.7974	6.273 Df - 78 P < 0.001***S
